# Assessment of Office-Based Probing with Dacryoendoscopy for Treatment of Congenital Nasolacrimal Duct Obstruction: A Retrospective Study

**DOI:** 10.3390/jcm12227048

**Published:** 2023-11-12

**Authors:** Yoshiki Ueta, Yuji Watanabe, Ryoma Kamada, Nobuya Tanaka

**Affiliations:** Department of Ophthalmology, Shinseikai Toyama Hospital, 89-10 Shimowaka, Imizu 939-0243, Toyama, Japan

**Keywords:** congenital nasolacrimal duct obstruction, dacryoendoscopy, epiphora, local anesthesia, office probing

## Abstract

We aimed to evaluate the safety and efficacy of office-based probing with dacryoendoscopy under local anesthesia for congenital nasolacrimal duct obstruction (CNLDO). This single-institution study retrospectively reviewed data on 72 eyes of 64 consecutive children (38 boys, 43 eyes; 26 girls, 29 eyes), aged between 6 and 17 (mean age: 10.0 ± 2.7) months with suspected CNLDO, from July 2016 to February 2022. These patients underwent probing with dacryoendoscopy under local anesthesia. CNLDO was diagnosed clinically based on the presence of epiphora and sticky eyes due to mucous discharge commencing within the first 3 months of life, increased tear meniscus height, and fluorescein dye disappearance test results. A total of 63 of the 72 eyes had narrowly defined CNLDO, and 9 eyes had other types of obstructions. The intervention success rate was 100% (63/63 eyes) for patients with typical CNLDO and 97.2% (70/72 eyes) for the entire study cohort. Moreover, CNLDO was classified into five types based on the features of the distal end of the nasolacrimal duct. Probing with dacryoendoscopy is safe and yields a high success rate in pediatric patients with CNLDO. This is the first study to assess the safety and efficacy of probing with dacryoendoscopy under local anesthesia in pediatric patients with CNLDO.

## 1. Introduction

Congenital nasolacrimal duct obstruction (CNLDO) is characterized by congenital membranous blockage of the distal end of the nasolacrimal duct [1]. The symptoms of CNLDO include epiphora or mucoid discharge from the eye within the first three months of life. Different variants of complex CNLDO exist, such as lacrimal punctum obstruction, agenesis of the lacrimal punctum, and bony obstruction. The incidence of CNLDO and lacrimal drainage dysfunction in infancy reportedly ranges from 6 to 20% [2,3,4].

Blind probing is the first choice of surgical treatment for CNLDO; however, the optimal timing of probing is being debated worldwide [5,6]. Spontaneous resolution of CNLDO by 12 months of age has been reported to occur in 82–96% of patients [2,7]. Some experts suggest that conservative treatment with eye drops and a lacrimal sac massage be recommended for a specific waiting period to allow spontaneous resolution, and upon failing, surgical treatment should be recommended under general anesthesia. However, set-up for general anesthesia is not normally available in most of the facilities, particularly for infants, and if available, it is labor-intensive for parents, doctors, and hospital personnel. Therefore, blind probing under local anesthesia can be considered in children aged <1 year in facilities where treatment under general anesthesia is not feasible. The CNLDO guidelines established in 2022 in Japan also recommend probing under local anesthesia for children 6–9 months of age [8].

Probing is a blind procedure, and canalicular stenosis has been shown to develop in pediatric patients after unsuccessful initial probings [9]. In recent years, several studies have evaluated dacryoendoscopy-guided probing for CNLDO [10,11,12,13,14,15,16,17], most of which were performed under general anesthesia. Since dacryoendoscopy allows for direct visualization of the lacrimal passage, this technique may facilitate safer and more reliable probing if performed under local anesthesia.

To the best of our knowledge, no previous study has examined the outcomes of probing with dacryoendoscopy, under local anesthesia in patients with CNLDO. Therefore, this is the first study that aimed to assess the safety and efficacy of this technique performed with local anesthesia in pediatric patients with CNLDO.

## 2. Materials and Methods

### 2.1. Ethics

This study was conducted in accordance with the tenets of the Declaration of Helsinki and approved by the Institutional Ethical Review Board of Shinseikai Toyama Hospital (approval number: 220223-1). We used an opt-out consent process by using the full written information about this research. Participants were included in the research unless their parents expressed their decision that they be excluded. The written full information was approved, and the requirement for obtaining informed consent was waived by the Institutional Review Board of Shinseikai Toyama Hospital.

### 2.2. Patients

We retrospectively reviewed the medical records of 72 eyes of 64 consecutive children (38 boys, 43 eyes; 26 girls, 29 eyes) aged between 6 and 17 (mean age: 10.0 ± 2.7) months with suspected CNLDO. These patients underwent probing with dacryoendoscopy under local anesthesia at our department between July 2016 and February 2022.

The clinical diagnosis of CNLDO was based on the presence of epiphora and sticky eye due to mucous discharge commencing within the first 3 months of life, increased tear meniscus height, and fluorescein dye disappearance test (FDDT) results. Additionally, the exclusion of a history of trichiasis, congenital glaucoma, keratitis, uveitis, and epidemic keratoconjunctivitis also assisted in establishing the clinical diagnosis of CNLDO. FDDT was performed as per the routine protocol [18]. Briefly, a drop of the fluorescein dye was instilled into the palpebral conjunctiva, and if the fluorescence persisted in the tear meniscus beyond 5 min, lacrimal drainage dysfunction was diagnosed. CNLDO might be ruled out if the fluorescent dye reached the nasal secretion due to the patency of the lacrimal duct. We did not perform lacrimal irrigation to diagnose CNLDO since it is an invasive procedure and uncomfortable for infants and children.

The parents of the children were instructed to perform lacrimal sac massage until 6 months of age, and we explained that they could request general anesthesia for their child if spontaneous resolution was not achieved until the age of 1 year or early probing under topical anesthesia. Probing was performed for patients for whom early intervention was requested. We limited patient enrollment to infants and young children who were not yet able to walk unaided.

### 2.3. Surgical Instruments

**Figure 1:**
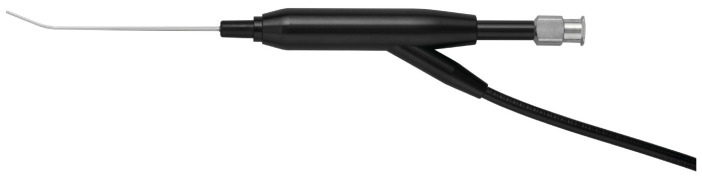
The MT-3000 dacryoendoscope (FiberTech Co., Tokyo, Japan) used in this study. This device has a tapered tip and acquires low-quality images (3000 pixels). The probe length is 50 mm, the outer diameter of the root is 1.0 mm, and the outer diameter of the tip is 0.7 mm, with an angulation of 27° 10 mm from the tip.

Dacryoendoscopy was performed using the MT-3000 device (FiberTech Co., Ltd., Tokyo, Japan) (Figure 1). The dacryoendoscope has a curved, rigid probe that contains a 3000-pixel fiberoptic bundle, illumination fibers, and irrigation channel. The probe length is 50 mm. The outer diameters of the root and tip measure 1.0 and 0.7 mm, respectively, with an angulation of 27° 10 mm from the tip. The MT-3000 possesses a tapered tip and yields images of low quality.

### 2.4. Surgical Procedure and Techniques

The surgical procedure employed in this study was as follows. A surgeon (Y.U.) skilled in dacryoendoscopic surgery performed the procedure, wherein ophthalmic anesthesia was induced via instillation of oxyprocaine and 4% lidocaine drops. Two caregivers restrained the patient with a retardation band. If the restraint was inadequate, the surgery was aborted. Insertion was achieved via the upper lacrimal punctum. The lower lacrimal punctum was used as the point of access in difficult cases. The lacrimal punctum was dilated using a punctal dilator, while the lacrimal sac was washed to eliminate as much pus as possible. The dacryoendoscope was inserted through the lacrimal punctum, and saline was injected to dilate the lacrimal canal while advancing the endoscope. Once the dacryoendoscope reached the lacrimal sac, the endoscope was held upright and advanced along the nasolacrimal duct to the distal end. The pus was then washed out to confirm the site of obstruction, followed by a puncture. If the obstruction site was difficult to confirm due to pus, a low-quality image, or body movement, semi-blind probing was attempted. Perforation was confirmed by assessing the nasal mucosa with the dacryoendoscope and by passing the saline solution. Antibiotic eye drops were administered for 3 days postoperatively. The children were followed up after 2 weeks to assess the patency of the lacrimal passage. Treatment success was defined as the disappearance of symptoms and a negative FDDT.

We have uploaded two videos of the procedure as supplementary material. Video S1 shows the dacryoendoscopic view of the patient with CNLDO in the left eye. The distal end of the nasolacrimal duct and the obstruction site are medial (Type 2). Video S2 shows the viewing of the dacryoendoscopic probing. The punctum is dilated and a dacryoendoscope is inserted, following which the obstruction site is perforated. It takes about 1–3 min from dilating the punctum to perforating and removing the dacryoendoscope. The Power Direct 365 program was used to create the videos.

### 2.5. Outcomes

The primary outcome was the rate of treatment success, defined as the disappearance of symptoms (epiphora and eye discharge) and a negative FDDT at 2 weeks post-surgery. The binomial proportion confidence intervals on the success rate were calculated for the 95% confidence interval (CI) using the Agresti–Coull method. The secondary outcomes included complications and endoscopic findings of the distal end of the nasolacrimal duct.

## 3. Results

The principal results of the study are presented in Table 1. A total of 63 of the 72 eyes had typical CNLDO (the term “typical” refers to congenital blockage of the distal end of the nasolacrimal duct, specifically the Hasner valve), and the remaining 9 eyes had other types of obstructions, including upper and lower lacrimal punctum obstruction (incomplete punctal canalization (IPC); *n* = 4). One of the four cases had IPC alone, two had combined distal end of the nasolacrimal duct obstruction, and one had combined inferior lacrimal canaliculus obstruction. The other five had common canaliculus obstruction (*n* = 1), lower nasolacrimal duct obstruction (*n* = 3), and lower nasolacrimal duct and inferior lacrimal canaliculus obstruction (*n* = 1).

The success rate of probing using dacryoendoscopy for typical CNLDO was 100% (63/63 eyes). Punctum obstruction was not successfully opened in one eye with upper and lower IPC and inferior lacrimal canaliculus obstruction. However, the duct was opened, and re-obstruction was observed in one case (common canaliculus obstruction) at the 2-week follow-up visit. Resolution was achieved in all other cases of CNLDO. The success rate of intervention in the entire study cohort was 97.2% (70/72 eyes). The binomial proportion confidence intervals on the success rate are 89.8–99.8% for the 95% CI (using the Agresti–Coull method). Seven eyes of six patients had undergone prior initial probing at other hospitals, all of which showed complete resolution. Restraint was performed safely on all children up to 17 months of age. There were no cases with complications such as damage to the punctum and canaliculus, hyperemia, infections, or false passage.

**Figure 2:**
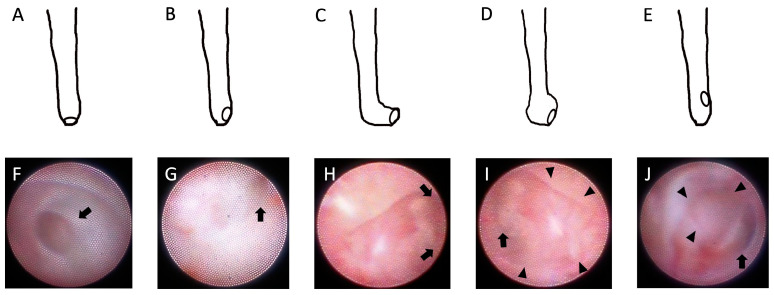
Classification of the distal end of the nasolacrimal duct by shape (illustrations and photographs). (**A**,**F**): Type 1, absence of dilation near the distal end of the nasolacrimal duct, and the obstructed valve of Hasner is located at the center (arrow). (**B**,**G**): Type 2, absence of dilation near the distal end of the nasolacrimal duct, and the obstructed valve of Hasner is medial (arrow). (**C**,**H**): Type 3, the distal end of the nasolacrimal duct is bent such that the obstructed site (beyond the arrow) cannot be seen from the front (irrespective of the presence or absence of dilation). (**D**,**I**): Type 4, presence of dilation near the distal end of the nasolacrimal duct (arrowheads), and the obstructed valve of Hasner is medial (arrow). (**E**,**J**): Type 5, presence of dilation near the distal end of the nasolacrimal duct (arrowheads), and the obstructed valve of Hasner is located in front of the nasal end (arrow). The small circles in (**A**–**E**) indicate the location of the obstruction, specifically the Hasner valve, with the nasal cavity located behind it. The CLIP STUDIO graphics program was used to create the illustration.

The obstruction site could not be observed on the endoscopic image in 31 (49%) eyes of the 63 cases of typical CNLDO due to the following reasons: first, the obstruction was punctured before visualization of the site of obstruction (semi-blind); second, the site of obstruction was difficult to visualize due to the presence of pus or body movement; and third, the resolution of the dacryoendoscope was poor. The occlusion site could be visualized in the remaining 32 cases. Thirty and two cases of simple membranous and stenosis-type obstructions, respectively, were observed. The eyes were classified into five groups based on the findings of the nasolacrimal duct distal end obstruction sites (Figure 2). Type 1 included eyes with no dilation near the distal end of the nasolacrimal duct and obstruction in the center (*n* = 2). Type 2 comprised eyes with no dilation near the distal end of the nasolacrimal duct, and the obstruction site was medial (*n* = 12). Type 3 included eyes in which the distal end of the nasolacrimal duct was bent such that the obstructed site could not be observed from the front (irrespective of the presence or absence of dilation) (*n* = 5). Type 4 was characterized by dilation near the distal end of the nasolacrimal duct, and the obstruction site was medial (*n* = 7). Type 5 was characterized by dilation near the distal end of the nasolacrimal duct, and the obstruction site was in front of the nasal end (probably due to dilation near the distal end of the nasolacrimal duct; *n* = 6).

## 4. Discussion

Herein, we report the first study to assess the safety and success rates of probing with dacryoendoscopy under local anesthesia in pediatric patients with CNLDO. The success rate of intervention in the entire patient population was 97.2% (70/72 eyes) and 100% in the eyes with typical CNLDO (63/63 eyes). The binomial proportion confidence intervals on the success rate are 89.8–99.8% for the 95% CI.

Fujimoto et al. [13] reported a 98.1% success rate with dacryoendoscopic probing in 54 CNLDO cases, including refractory disease. Matsumura et al. [14] also reported a 100% success rate for dacryoendoscopic probing under general anesthesia in 56 cases. The success rate of blind probing is 75–92% [5,19,20,21,22,23,24], and if limited to office probing, the success rate is 75–88.6% [21,22,23,24]. In comparison, dacryoendoscopic probing may have a relatively higher success rate.

The site of obstruction could be visualized in 32 cases, and based on this, CNLDO was classified into five types. It is not difficult to perforate the obstruction in types 1 and 2, even with blind probing. However, puncturing is difficult with normal blind probing in types 3 to 5 because the blockage is not located at the distal end of the duct. It may even be impossible to penetrate the blockage in types 3 and 5 without using dacryoendoscopy. Matsumura et al. [14] reported that the precise location of the blockage in the simple type was approximately 0.5–1.0 mm proximal to the end of the duct. In addition, the duct sometimes ends with a “pocket” in the nasal mucosa of the lateral wall of the nasal cavity. This particular anatomical configuration could be considered as a factor contributing to the failure of probing using the blind technique. It is thought that the failure rate of blind probing may be high in types 3, 4, and 5; the use of dacryoendoscopy can prevent misdirected probing, thus improving the success rate. Furthermore, dilation near the distal end of the nasolacrimal duct was another feature that could be visualized using dacryoendoscopy. It is unclear whether this dilation was an original feature or an abnormal manifestation of chronic inflammation. Given that distal end dilation is frequently absent in adult dacryoendoscopic observations, this dilation may be a consequence of chronic inflammation that tends to revert to its characteristic shape with the subsidence of the inflammation. The greater the severity of the dilation, the greater the degree of displacement of the obstruction site. It has been reported that the success rate of interventions decreases with increasing age [20]. This may be attributed to changes in the shape of the distal end of the nasolacrimal duct due to chronic inflammation.

We used the MT-3000 device (FiberTech Co., Ltd., Tokyo, Japan) as the dacryoendoscope, which had a curved, rigid probe containing a 3000-pixel fiberoptic bundle. The outer diameters of the root and tip measured 1.0 and 0.7 mm, respectively, with an angulation of 27°, 10 mm from the tip. The MT-3000 features a tapered tip but yields low-quality images. Conventional dacryoendoscopes typically employ a straight-type probe, but in Japan, a bent-type probe has been developed [8]. In terms of resolution rate, five studies [10,11,13,14,15] that used a curved dacryoendoscope have reported success rates that are fairly consistent across the studies (ranging from 92.3% to 100%). On the other hand, among the three studies [12,16,17] that utilized a straight dacryoendoscope, success rates varied from 53.8% to 94.4%. While there are no randomized control trials that investigate the impact of the shape of the handpiece of a dacryoendoscope on success rates, there is a tendency for the success rate to be higher when curved dacryoendoscopes are used. Anatomically, the lacrimal duct is curved, and curved dacryoendoscopes can visualize these lacrimal ducts more accurately. Hence, we believe that using a bent-type dacryoendoscope is preferable for endoscopic probing of the lacrimal duct. Additionally, the MT-3000 has a tapered tip and provides low-quality images. Better resolution is essential for effectively identifying obstruction sites. In some cases, in our study, the obstruction site was difficult to identify due to this low resolution. However, in children, the lacrimal punctums are smaller than in adults. Furthermore, when operating under local anesthesia, it is desirable to use a less invasive technique to keep the duration of the procedure as short as possible. Consequently, we prioritized ease of insertion and used a dacryoendoscope with a tapered 0.7 mm tip. When dacryoendoscopic probing is performed under general anesthesia, it is advisable to employ a dacryoendoscope with a higher resolution.

Compared with blind probing, dacryoendoscopic probing requires preparation for dacryoendoscopy, skill, and larger lacrimal punctum dilation and has no advantage for upper lacrimal system obstruction. In addition, it is not a substitute for dacryoendoscopic probing under general anesthesia because of the age limitation and the inability to insert a lacrimal tube. However, dacryoendoscopic probing under topical anesthesia is a short procedure that usually takes 2–3 min, from dilating the punctum to perforating and removing the dacryoendoscope, and nasolacrimal duct visualization can be achieved. The advantages of dacryoendoscopic probing over blind probing under local anesthesia include the ability to (1) reach the distal end of the nasolacrimal duct without creating a false pathway, (2) respond to the shape of the distal end of the nasolacrimal duct, and (3) find and treat obstructions in areas other than the distal end of the nasolacrimal duct (such as upper or lower nasolacrimal duct obstruction and dacryolith). Moreover, dacryoendoscopic probing under general anesthesia is limited due to a lack of appropriate facilities that can provide general anesthesia in children. Furthermore, general anesthesia is expensive and associated with complications. Therefore, office-based dacryoendoscopic probing under topical anesthesia may be an alternative to conventional blind probing and dacryoendoscopic probing under general anesthesia.

This study has several limitations. First, it incorporated a retrospective case series design, and there is no control group like blind probing. Second, patients were followed up for only 2 weeks after surgery. Subsequent re-occlusion might have occurred, which was not evaluated in this study. However, once the correct duct is opened, re-occlusion is unlikely to occur in patients with typical CNLDO [20,25]. Therefore, we believe that follow-up examinations 2 weeks postoperatively are sufficient if accurate puncturing is ensured during surgery. Third, our results may not be generalizable to all clinicians because the operation of the dacryoendoscopy requires suitable skills. Fourth, since families that select general anesthesia may have risk factors for failure in the clinic, the study’s opt-out design may induce bias toward favorable results. Within the scope of these limitations, this study demonstrated the safety and high success rate of office probing with dacryoendoscopy. Therefore, this technique should be considered at facilities where general anesthesia is not available.

## 5. Conclusions

Probing with dacryoendoscopy has a high success rate and can be considered as a safe treatment modality for CNLDO. Although it is necessary to become proficient with the instruments and handling of the dacryoendoscopy, this technique may be recommended at facilities where general anesthesia is not available.

## Figures and Tables

**Table 1:** Comparative results of the obstruction site, sample size, and success rate.

Obstruction Site	*n*	Success Rate
Distal end of the nasolacrimal duct alone (typical CNLDO)	63	100% (63/63)
Upper and lower lacrimal punctum (IPC)		
IPC alone	1	100% (1/1)
Distal end of the nasolacrimal duct	2	100% (2/2)
Inferior lacrimal canaliculus	1	0% (0/1)
Common canaliculus	1	0% (0/1)
Lower nasolacrimal duct	3	100% (3/3)
Inferior lacrimal canaliculus and lower nasolacrimal duct	1	100% (1/1)
Total	72	97.2% (70/72)

CNLDO, congenital nasolacrimal duct obstruction; IPC, incomplete punctal canalization.

## Data Availability

All data analyzed in this study are included in this article. Further inquiries can be directed to the corresponding author.

## Supplementary Materials

The following supporting information can be downloaded at: https://www.mdpi.com/article/10.3390/jcm12227048/s1, Video S1: dacryoendoscopic view; Video S2: View of dacryoendoscopic probing.

## Abbreviations

CNLDO	congenital nasolacrimal duct obstruction
FDDT	fluorescein dye disappearance test
IPC	incomplete punctal canalization
